# Frequency and determinants of use of immunosuppressants in the Australian Scleroderma Cohort Study

**DOI:** 10.1177/23971983251342690

**Published:** 2025-05-22

**Authors:** Jessica L Fairley, Dylan Hansen, Susanna Proudman, Joanne Sahhar, Gene-Siew Ngian, Diane Apostolopoulos, Jennifer Walker, Lauren V Host, Wendy Stevens, Laura Ross, Mandana Nikpour

**Affiliations:** 1 The University of Melbourne, Melbourne, VIC, Australia; 2 Department of Rheumatology, St. Vincent’s Hospital Melbourne, Fitzroy, VIC, Australia; 3 The University of Adelaide, Adelaide, SA, Australia; 4 Royal Adelaide Hospital, Adelaide, SA, Australia; 5 Monash Health, Melbourne, VIC, Australia; 6 Monash University, Melbourne, VIC, Australia; 7 Flinders University, Bedford Park, SA, Australia; 8 Fiona Stanley Hospital, Perth, WA, Australia; 9 School of Public Health, The University of Sydney, Sydney, NSW, Australia; 10 Royal Prince Alfred Hospital, Sydney, NSW, Australia; 11 SydneyMSK Research Flagship Centre, The University of Sydney, Sydney, NSW, Australia

**Keywords:** Systemic sclerosis, immunosuppressant, corticosteroid, non-corticosteroid immunosuppressants, time trends

## Abstract

**Objectives:**

To assess the frequency and determinants of immunosuppressant medication use in systemic sclerosis and changes in prescribing patterns over time.

**Methods:**

The Australian Scleroderma Cohort Study participants meeting the American College of Rheumatology/European Alliance of Associations for Rheumatology criteria for systemic sclerosis with recorded treatment data were included. The Chi-square, two-sample *t*-tests or Wilcoxon rank-sum tests were used for between-group comparison as appropriate. Multivariable logistic regression models were used to establish the determinants of the use of immunosuppressants.

**Results:**

Of 2019 participants, 60% received immunosuppressants, including 81% of those with diffuse systemic sclerosis and 52% of those with limited systemic sclerosis (p < 0.001). Forty-six percent of patients received prednisolone and 40% disease-modifying anti-rheumatic drugs. Immunosuppressant use was more common in those with severe or inflammatory systemic sclerosis features, including interstitial lung disease, synovitis or myositis. Comparing prescribing patterns early in incident systemic sclerosis from 2007–2014 to 2015–2024, disease-modifying anti-rheumatic drug use increased (35% vs 56%, p < 0.001), while prednisolone use decreased (24% vs 17%, p = 0.046). Immunosuppressants were commenced earlier in incident systemic sclerosis in 2015–2024 versus 2007–2014 (1.8 (interquartile range = 1.0–3.2) vs 2.4 (interquartile range = 1.2–4.0) years, p = 0.011). In multivariable modelling, prednisolone use was associated with diffuse systemic sclerosis (odds ratio = 1.8, 95% confidence interval = 1.4–2.2, p < 0.001), interstitial lung disease (odds ratio = 2.1, 95% confidence interval = 1.7–2.5, p < 0.001), myositis (odds ratio = 2.7, 95% confidence interval = 1.8–4.0, p < 0.001), synovitis (odds ratio = 2.2, 95% confidence interval = 1.8–2.6, p < 0.001) and systemic sclerosis heart involvement (odds ratio = 1.4, 95% confidence interval = 1.0–2.0, p = 0.044). Disease-modifying anti-rheumatic drug exposure was associated with diffuse systemic sclerosis (odds ratio = 2.7, 95% confidence interval = 2.1–3.4, p < 0.001), interstitial lung disease (odds ratio = 2.2, 95% confidence interval = 1.7–2.7, p < 0.001), myositis (odds ratio = 3.6, 95% confidence interval = 2.4–5.5, p < 0.001) and synovitis (odds ratio = 4.2, 95% confidence interval = 3.5–5.2, p < 0.001) and inversely associated with age (odds ratio = 0.7, 95% confidence interval = 0.5–0.8, p < 0.01) and pulmonary arterial hypertension (odds ratio = 0.5, 95% confidence interval = 0.4–0.7, p < 0.001). In subgroups with diffuse systemic sclerosis and limited systemic sclerosis and different autoantibody profiles, findings were generally similar, with interstitial lung disease, synovitis and myositis tending to be associated with prednisolone and/or disease-modifying anti-rheumatic drug use, as was systemic sclerosis heart involvement in diffuse systemic sclerosis (p = 0.038).

**Conclusion:**

Immunosuppressant use is common in systemic sclerosis, with broadly similar determinants of usage among subtypes and autoantibody status. These real-world data suggest that disease-modifying anti-rheumatic drug use has increased, with earlier implementation of treatment, and a reduction in use of glucocorticoids.

## Introduction

In systemic sclerosis (SSc), only 30%–40% of patients may receive immunosuppressants,^1,2^ compared to over 85% of those with systemic lupus erythematosus (SLE).^3^ This is likely in part due to the complex pathological triad of fibrosis, vasculopathy and inflammation underlying SSc, meaning that not all disease features are effectively treated with immunosuppressants. Furthermore, the toxicity of some agents (e.g. cyclophosphamide) has limited their widespread use despite their efficacy in some disease manifestations.^4^ Some agents also confer risk of SSc-specific complications, for example, SSc renal crisis (SRC) with glucocorticoid use.^5,6^

The emergence of newer agents with an improved safety profile,^4^ along with further data supporting a larger role for immunosuppressants in the treatment of several SSc features, has led to more widespread use of immunosuppressants in a broader patient cohort.^7^ The wider literature suggests a particular increase in use among those with early diffuse systemic sclerosis (dcSSc),^2^ with autoantibody status being an important predictor of immunosuppressant use.^2^ However, limited real-world data exist in large cohorts describing prescribing patterns and determinants of use of immunosuppressants in SSc including in subgroups with limited systemic sclerosis (lcSSc) and dcSSc and different autoantibody profiles. Accordingly, we performed this study to identify the frequency and determinants of immunosuppressant use in SSc and compare immunosuppressant use between subgroups by disease class and autoantibody status, as well as to describe any change in patterns of use over time.

## Methods

Participants were recruited from the multicentre, prospective Australian Scleroderma Cohort Study (ASCS). Participants meeting the American College of Rheumatology/European Alliance of Associations for Rheumatology (ACR/EULAR) criteria for SSc^8^ recruited between 2007 and December 2024 and were included if medication use data were recorded at ⩾1 study visit. The ASCS has been approved by all human research ethics committees at participating sites with St Vincent’s Hospital Melbourne acting as the coordinating site (HREC-A 020/07). Written informed consent was obtained from all participants.

### Definition of immunosuppressant exposure

Medication use was recorded at each annual study visit from patient-report and medical record review. Exposure to each agent was recorded as ‘current’, ‘previous’ or ‘never’. Indication for medication, dose and duration of use are not recorded within the ASCS. Immunosuppressant use was defined as exposure to prednisolone, hydroxychloroquine, conventional synthetic disease-modifying anti-rheumatic drugs (csDMARDs) or biologic DMARDs (bDMARDs) as well as intravenous immunoglobulin (IVIG) or cyclophosphamide. Disease-modifying anti-rheumatic drug (DMARD) use was defined as ever using csDMARD or bDMARDs. csDMARDs recorded were azathioprine, methotrexate, mycophenolate, calcineurin inhibitors and leflunomide. bDMARDs included abatacept, tumour necrosis factor (TNF)-alpha inhibitors, tocilizumab and/or rituximab. Frequency of stem cell transplantation (SCT) was recorded but not included in the definition of immunosuppressant exposure. Combination immunosuppressant use was defined as those recording ‘current’ use for either methotrexate and mycophenolate or methotrexate and a biologic agent at the same study visit.

To identify change in immunosuppressant prescription over time, we examined the early use of immunosuppressants (within first 5 study visits) in those with incident SSc (⩽5 years SSc duration at recruitment). We separated participants into two epochs: those recruited between 2007 and 2014 and those recruited from 2015 to 2024. We examined the frequency of DMARD use, as well as hydroxychloroquine, IVIG and cyclophosphamide between groups. We compared prednisolone use between groups, as well as the number of visits at which prednisolone was prescribed early in follow-up as a measure of overall prednisolone exposure.

### Disease features and comorbidities

Demographic and disease data were collected annually. Disease manifestations and autoantibody results were considered present if they had ever been reported since SSc diagnosis. Disease onset was defined as the date of onset of the first non-Raynaud’s phenomenon SSc manifestation. The LeRoy criteria were used to determine disease subtype: dcSSc or lcSSc.^9^ Incident SSc was defined as ASCS recruitment within 5 years of SSc onset. The presence of gastrointestinal involvement and comorbidities including malignancy was recorded from patient-reported history and medical record review at each study visit. Ischaemic heart disease (IHD) was defined as patient-reported angina or myocardial infarction, coronary artery bypass grafting or stenting. Pulmonary arterial hypertension (PAH) was defined by right heart catheterisation findings according to the revised PAH classification criteria^10^ or previous classification criteria.^11^ Interstitial lung disease (ILD) was diagnosed using high-resolution computed tomography (HRCT) of the chest performed at physician discretion in response to clinical assessment or abnormal pulmonary function test results. A transthoracic echocardiogram including measurement of left ventricular ejection fraction (LVEF) was performed annually as part of PAH screening. SRC was diagnosed in the presence of two of the three criteria: new-onset hypertension with no alternate cause, unexplained rise in serum creatinine or microangiopathic haemolytic anaemia. SSc heart involvement (SHI) was determined by physician assessment based on the presence of systolic or diastolic dysfunction or rhythm disturbance attributable to SSc. Myositis was identified by the treating clinician based on positive biopsy or elevated creatine kinase combined with abnormal magnetic resonance imaging or electromyography findings. Overlap rheumatological conditions were diagnosed by the treating clinician without mandating that patients fulfil additional diagnostic criteria. C-reactive protein (CRP) elevation was defined as >5 mg/L.

### Statistical analysis

Characteristics of study participants are presented as mean (standard deviation (SD)) for normally distributed continuous variables, median (interquartile range (IQR)) for non-normally distributed continuous variables and as a number (percentage) for discrete variables. Comparisons between those who had ever received immunosuppressants and those who had not were performed using the two-sample *t*-test for normally distributed continuous variables, the Wilcoxon rank-sum test for non-normally distributed continuous variables and the chi-square test for discrete variables. Logistic regression was used to determine the associations of immunosuppressant exposure ever from ASCS recruitment for each of prednisolone and DMARD use. Covariates for the multivariable models were selected based on clinical and statistical significance and were not collinear. Prespecified subgroups included dcSSc, lcSSc and autoantibody positivity (anticentromere antibodies (ACAs), Scl-70 or RNA polymerase-3 antibodies). Results are presented as odds ratios (ORs) with 95% confidence intervals (CIs). p-values of <0.05 were considered statistically significant. Analysis was performed using STATA 17.0 (StataCorp LP, College Station, TX, USA).

## Results

Among 2019 participants with a median age of 47.4 (IQR = 36.5–57.4) years, 15% were male and 28% had dcSSc (Table 1). One-thousand, two hundred and eighteen participants received immunosuppression (60%), including 46% receiving prednisolone and 40% DMARDs (Table 2). Participants receiving immunosuppressants had been exposed to a median of 2 agents (IQR = 1–3). Among the cohort, 23% received hydroxychloroquine, 39% other csDMARDs, 8% cyclophosphamide, 1% IVIG and 4% bDMARDs. Among csDMARDs, the most common was methotrexate (24%), followed by mycophenolate (15%) and azathioprine (8%), with leflunomide (1%) and calcineurin inhibitors (2%) less commonly used. bDMARD use was relatively uncommon, with rituximab the most prescribed (2%), followed by TNF-α inhibitors (1%) and tocilizumab (1%).

**Table 1.** Disease features of the cohort, comparing those ever exposed to immunosuppressants, and unexposed participants.

Variable^a^	Overall cohort (n = 2019)	Immunosuppressant-exposed^b,c^ (n = 1218, 60.3%)	Immunosuppressant-naïve (n = 801, 39.7%)	p
Age at SSc onset (years) (n = 1887)	47.4 (36.5-57.4)	47.4 (36.0-56.9)	47.3 (37.3-58.0)	0.381
Male sex	298 (14.8%)	203 (16.7%)	95 (11.9%)	0.003
Diffuse cutaneous SSc	555 (27.5%)	451 (37.0%)	104 (13.0%)	< 0.001
Non-Caucasian ethnicity (n = 1899)	172 (9.1%)	121 (10.4%)	51 (6.9%)	0.009
Disease duration at recruitment (years) (n = 1886)	7.1 (2.5-15.7)	6.4 (2.1-14.9)	8.9 (3.3-17.5)	< 0.001
Follow-up (years)	4.3 (1.3-8.6)	4.8 (1.7-9.0)	3.6 (1.0-7.7)	< 0.001
Overlap features (n = 1267)	182 (14.4%)	155 (19.5%)	27 (5.7%)	< 0.001
Inflammatory arthritis (n = 1267)	77 (6.1%)	74 (9.3%)	3 (0.6%)	< 0.001
SLE (n = 1267)	31 (2.4%)	26 (3.3%)	5 (1.1%)	0.014
Polymyositis/dermatomyositis (n = 1267)	36 (2.8%)	32 (4.0%)	4 (0.9%)	0.001
Sjogren syndrome (n = 1267)	52 (4.1%)	36 (4.5%)	16 (3.4%)	0.329
MRSS (highest)^b^ (n = 1968)	8 (4-16)	10 (5-20)	7 (4-11)	< 0.001
Malignancy^b^	486 (24.1%)	304 (25.0%)	182 (22.7%)	0.250
ANA centromere^b^ (n = 1951)	904 (46.3%)	395 (33.6%)	509 (65.5%)	< 0.001
ENA^b^				
Scl70 (n = 1917)	290 (15.1%)	229 (19.7%)	61 (8.1%)	< 0.001
Ro (n = 1917)	190 (9.9%)	130 (11.2%)	60 (7.9%)	0.020
La (n = 1913)	37 (1.9%)	26 (2.2%)	11 (1.5%)	0.223
U1RNP (n = 1915)	127 (6.6%)	104 (9.0%)	23 (3.0%)	< 0.001
Scl/PM (n = 1911)	39 (2.0%)	34 (2.9%)	5 (0.7%)	0.001
RNA polymerase-3^b^ (n = 1381)	197 (14.3%)	144 (16.5%)	53 (10.4%)	0.002
Ischaemic heart disease^b,d^ (n = 2005)	281 (14.0%)	184 (15.2%)	97 (12.2%)	0.056
SHI^b,e^	183 (9.1%)	134 (11.0%)	49 (6.1%)	< 0.001
LVEF < 50%^b^ (n = 1781)	98 (5.5%)	72 (6.7%)	26 (3.7%)	0.007
PAH^b^	231 (11.4%)	136 (11.2%)	95 (11.9%)	0.632
ILD (on HRCT)^b,f^	578 (28.6%)	445 (36.5%)	133 (16.6%)	< 0.001
Digital ulcers^b^	1,071 (53.0%)	679 (55.7%)	392 (48.9%)	0.003
Raynaud’s phenomenon^b^	2,000 (99.1%)	1,205 (98.9%)	795 (99.3%)	0.469
Tendon friction rubs^b^ (n = 1978)	178 (9.0%)	137 (11.4%)	41 (5.3%)	< 0.001
Synovitis^b^ (n = 2004)	841 (42.0%)	636 (52.6%)	205 (25.8%)	< 0.001
SRC^b^	72 (3.6%)	52 (4.3%)	20 (2.5%)	0.036
Gastrointestinal involvement^b,g^	1,900 (94.1%)	1,162 (95.4%)	738 (92.1%)	0.002
Myositis^b,h^	149 (7.4%)	135 (11.1%)	14 (1.7%)	< 0.001
CRP > 5 mg/L^b^ (n = 1914)	1,011 (52.8%)	692 (58.8%)	319 (43.2%)	< 0.001

ANA: antinuclear antibody; CK: creatine kinase; CRP: C-reactive protein; dcSSc: diffuse cutaneous systemic sclerosis; DLCO: diffusing capacity for carbon monoxide; DMARD: disease-modifying anti-rheumatic drug; ENA: extractable nuclear antigen; FVC: forced vital capacity; ILD: interstitial lung disease; IVIG: intravenous immune globulin; LVEF: left ventricular ejection fraction; mm/hr: millimetres per hour; mm Hg: millimetres of mercury; mg/L: milligramme per litre; MRSS: Modified Rodnan Skin Score; PAH: pulmonary arterial hypertension; RVSP: right ventricular systolic pressure; SHI: SSc Heart Involvement; SSc: systemic sclerosis; TNF (tumour necrosis factor).

Data presented for whole cohort (n = 2019) unless otherwise specified.

a Number and percentage or median/interquartile range presented as appropriate.

b Ever from SSc onset. If value at first visit not available, first recorded value within 5 years of recruitment presented.

c Immunosuppressant exposure defined as any immunosuppressive or immunomodulatory therapy including glucocorticoids, IVIg, synthetic or biologic disease-modifying therapy.

d Ischaemic heart disease defined as patient-reported angina/myocardial infarction or abnormal coronary angiography.

e SHI was determined by physician assessment based on the presence of systolic or diastolic dysfunction or rhythm disturbance attributable to SSc.

f ILD diagnosed on high-resolution CT (HRCT) chest.

g Gastroinestinal involvement defined as ever experiencing upper (reflux symptoms, Barret’s oesophagus, oesophageal dysmotility, oesophageal stricture, dysphagia, vomiting or gastric antral vascular ectasia) or lower gastrointestinal involvement (bowel dysmotility, pseudo-obstruction, faecal incontinence, constipation, diarrhoea or bloating).

h Myositis was identified by the treating clinician based on positive biopsy or elevated CK combined with abnormal MRI or EMG findings.

**Table 2.** Description of immunosuppressant prescription in the cohort overall and in those with diffuse and limited cutaneous SSc.

	Whole cohort (n = 2019, 100%)	dcSSc (N = 555, 27.5%)	lcSSc (N = 1464, 72.5%)	p
Any immunosuppressant exposure^a,b^	1,218 (60.3%)	451 (81.3%)	767 (52.4%)	< 0.001
Prednisolone^a^	926 (45.9%)	335 (60.4%)	591 (40.4%)	< 0.001
Hydroxychloroquine^a^	473 (23.4%)	137 (24.7%)	336 (23.0%)	0.412
Any DMARD use^a,c^	802 (39.7%)	336 (60.5%)	466 (31.8%)	< 0.001
Conventional synthetic DMARD^a,d^	785 (38.9%)	328 (59.1%)	457 (31.2%)	< 0.001
Azathioprine^a^	162 (8.0%)	59 (10.6%)	103 (7.0%)	0.008
Calcineurin inhibitor^a^	36 (1.8%)	14 (2.5%)	23 (1.6%)	0.155
Leflunomide^a^	29 (1.4%)	2 (0.4%)	27 (1.8%)	0.012
Methotrexate^a^ (n = 2018)	488 (24.2%)	224 (40.4%)	264 (18.0%)	< 0.001
Mycophenolate^a^	304 (15.1%)	201 (36.2%)	103 (7.0%)	< 0.001
Biologic DMARD^a,e^	74 (3.7%)	34 (6.1%)	40 (2.7%)	< 0.001
Abatacept^a^	9 (0.4%)	2 (0.4%)	7 (0.5%)	0.723
TNF-alpha inhibitor^a^	21 (1.0%)	3 (0.5%)	18 (1.2%)	0.173
Tocilizumab^a^	20 (1.0%)	11 (2.0%)	9 (0.6%)	0.006
Rituximab^a^	41 (2.0%)	25 (4.5%)	16 (1.1%)	< 0.001
IVIG^a^	28 (1.4%)	16 (2.9%)	12 (0.8%)	< 0.001
Cyclophosphamide^a^	165 (8.2%)	103 (18.6%)	62 (4.2%)	< 0.001
Stem cell transplantation^a^ (n = 1992)	6 (0.3%)	6 (1.1%)	0 (0.0%)	< 0.001
Combination therapies^a^				
Methotrexate and mycophenolate (n = 2018)^a^	24 (1.2%)	19 (3.4%)	5 (0.3%)	< 0.001
Methotrexate and biologic agent (n = 2018)^a^	25 (1.2%)	8 (1.4%)	17 (1.2%)	0.612

csDMARD: conventional synthetic disease-modifying anti-rheumatic drug; DMARD: disease-modifying anti-rheumatic drug; dcSSc: diffuse SSc; IVIG: intravenous immune globulin; lcSSc: limited SSc; TNF: tumour necrosis factor.

Data presented for whole cohort (n = 2019) unless otherwise specified.

a Denotes ever from SSc onset.

b Denotes use of any glucocorticoid or non-glucocorticoid immunosuppressant, including biologics, IVIG and cyclophosphamide.

c Denotes any use of either conventional synthetic or biologic DMARD ever.

d Conventional synthetic DMARDs include azathioprine, methotrexate, mycophenolate, calcineurin inhibition and leflunomide.

e Biologic agents include abatacept, TNF-alpha inhibitors, tocilizumab or B-cell depletion.

### Description of those receiving immunosuppressants

Immunosuppressant-exposed participants were more likely to be men with dcSSc and higher peak modified Rodnan skin score (MRSS) (both p < 0.01) (Table 1). Immunosuppressant-exposed participants were more likely to be of non-Caucasian ethnicity (p = 0.02), with a shorter SSc duration at recruitment (p < 0.01) but longer follow-up (p < 0.01). Immunosuppressant-exposed participants were more likely to have overlap features (including rheumatoid arthritis, SLE, myositis and Sjogren Syndrome, p < 0.01). This group were more likely to have Scl70 and RNA polymerase-3 antibodies (both p < 0.01) and less likely to have ACA positivity (p < 0.01). Immunosuppressant-exposed participants were more likely to have anti-Ro, U1RNP and Scl/PM antibodies (p < 0.05).

Severe SSc features were more common in those receiving immunosuppressants, including SHI, ILD, synovitis and myositis (p < 0.05). Cardiac disease was also more common in this group, including depressed LVEF and IHD (p < 0.01). There was no difference in the frequency of PAH. Digital ulcers, tendon friction rubs and SRC were more common in those receiving immunosuppressants (p < 0.05), as was raised CRP (p < 0.01) and gastrointestinal involvement (p < 0.01). There was no difference in frequency of malignancy between immunosuppressant-exposed and unexposed participants (p = 0.25).

### Clinical features of participants receiving immunosuppression by SSc duration

Those with SSc duration at ASCS recruitment < 7 years (cohort median) were more likely to receive immunosuppression than those with an SSc duration ⩾ 7 years (64.8% vs 57.1%, p = 0.001) (Supplementary Table S1). Of participants receiving immunosuppression (n = 1149), those with a longer SSc duration at recruitment were more likely to be older women with lcSSc (p < 0.001) and of Caucasian ethnicity (p = 0.010). Those with longer SSc duration were more likely to have overlap features (p = 0.013), antinuclear antibody (ANA) centromere positivity (p < 0.001) and were less likely to have Scl-70, Scl/Pm or RNA polymerase-3 positivity (all p < 0.050). Those with longer SSc duration were more likely to have IHD and PAH (both p = 0.005) and less likely to have tendon friction rubs (p = 0.030).

### Immunosuppressant prescribing in those with dcSSc and lcSSc

Among those with dcSSc, 81% had received immunosuppressants compared to 52% of those with lcSSc (p < 0.001) (Table 2). Prednisolone was prescribed in 60% of dcSSc compared to 40% of lcSSc (p < 0.001), while DMARDs were prescribed in 61% of dcSSc versus 32% of lcSSc (p < 0.001) (Table 2). The use of all csDMARDs was more common in those with dcSSc, except for leflunomide which was more common in lcSSc (p = 0.012) and calcineurin inhibition which was similar between groups (p = 0.155). Combination treatment with methotrexate and mycophenolate was also more common in dcSSc (p < 0.001). bDMARDs were more commonly used in dcSSc (p < 0.001), particularly rituximab (p < 0.001) and tocilizumab (p = 0.006), as were cyclophosphamide (p < 0.001) and IVIG (p < 0.001). Stem-cell transplantation was only performed in those exposed to other immunosuppressants (p < 0.001).

### Associations of prednisolone and DMARD exposure

#### Overall cohort

In the cohort overall, prednisolone use was associated with dcSSc (OR = 1.8, 95% CI = 1.4–2.2, p < 0.001), ILD (OR = 2.1, 95% CI = 1.7–2.5, p < 0.001), myositis (OR = 2.7, 95% CI = 1.8–4.0, p < 0.001), synovitis (OR = 2.2, 95% CI = 1.8–2.6, p < 0.001) and SHI (OR = 1.4, 95% CI = 1.0–2.0, p = 0.044) as well as gastrointestinal involvement, although not meeting statistical significance (OR = 1.5, 95% CI = 1.0–2.3, p = 0.062) (Table 3; univariable analyses in Supplementary Table S3). DMARD exposure was associated with dcSSc (OR = 2.7, 95% CI = 2.1–3.4, p < 0.001), ILD (OR = 2.2, 95% CI = 1.7–2.7, p < 0.001), myositis (OR = 3.6, 95% CI = 2.4–5.5, p < 0.001) and synovitis (OR = 4.2, 95% CI = 3.5–5.2, p < 0.01), as well as gastrointestinal involvement, although not meeting statistical significance (OR = 1.6, 95% CI = 1.0–2.5, p = 0.063) (Table 3; univariable analyses in Supplementary Table S3). DMARD exposure was inversely associated with age (OR = 0.7, 95% CI = 0.5–0.8, p < 0.001) and PAH (OR = 0.5, 95% CI = 0.4–0.7, p < 0.001).

**Table 3.** Multivariable logistic regression models for exposure to prednisolone or disease-modifying anti-rheumatic drugs (biologic or conventional synthetic) ever in the overall cohort and in subgroups by disease class and autoantibody status.

Cohort overall (n = 2002)	Model 1: associations of prednisolone exposure			Model 2: associations of DMARD exposure^a^		
	OR	95%CI	p	OR	95%CI	p
Age older than cohort median at SSc onset (N = 46.8 years^b^)	1.0	0.8-1.2	0.652	0.7	0.5-0.8	< 0.001
Male sex	1.1	0.8-1.4	0.620	1.2	0.9-1.7	0.133
Diffuse SSc	1.8	1.4-2.2	< 0.001	2.7	2.1-3.4	< 0.001
PAH^c^	1.0	0.7-1.4	0.981	0.5	0.4-0.7	< 0.001
ILD^c,d^	2.1	1.7-2.5	< 0.001	2.2	1.7-2.7	< 0.001
Myositis^c,e^	2.7	1.8-4.0	< 0.001	3.6	2.4-5.5	< 0.001
Synovitis^c^	2.2	1.8-2.6	< 0.001	4.2	3.5-5.2	< 0.001
Gastrointestinal involvement^c,f^	1.5	1.0-2.3	0.062	1.6	1.0-2.5	0.063
SHI^c,g^	1.4	1.0-2.0	0.044	1.2	0.8-1.7	0.445
SRC^c^	1.4	0.8-2.3	0.244	1.1	0.6-1.9	0.797
dcSSc only (n = 550)	OR	95%CI	p	OR	95%CI	p
Age older than cohort median at SSc onset (N = 46.8 years^b^)	0.8	0.6-1.2	0.346	0.5	0.3-0.7	< 0.001
Male sex	1.0	0.7-1.6	0.864	1.1	0.7-1.6	0.785
PAH^c^	1.8	1.0-3.5	0.065	0.7	0.4-1.3	0.285
ILD^c,d^	1.4	1.0-2.1	0.059	2.1	1.4-3.1	< 0.001
Myositis^c,e^	1.4	0.8-2.5	0.280	2.4	1.2-4.6	0.012
Synovitis^c^	1.6	1.1-2.3	0.008	2.5	1.7-3.7	< 0.001
Gastrointestinal involvement^c,f^	2.1	0.9-4.9	0.089	1.6	0.7-3.7	0.306
SHI^c,g^	1.9	1.0-3.4	0.038	1.1	0.6-2.0	0.743
SRC^c^	1.3	0.7-2.5	0.431	1.0	0.5-1.8	0.916
lcSSc only (n = 1452)	OR	95%CI	p	OR	95%CI	p
Age older than cohort median at SSc onset (N = 46.8 years^b^)	1.0	0.8-1.2	0.942	0.8	0.6-1.0	0.039
Male sex	1.1	0.8-1.5	0.683	1.4	1.0-2.1	0.075
PAH^c^	0.9	0.6-1.2	0.376	0.4	0.3-0.7	< 0.001
ILD^c,d^	2.4	1.8-3.1	< 0.001	2.2	1.6-2.9	< 0.001
Myositis^c,e^	4.3	2.5-7.3	< 0.001	4.6	2.7-7.9	< 0.001
Synovitis^c^	2.4	2.0-3.1	< 0.001	5.2	4.1-6.7	< 0.001
Gastrointestinal involvement^c,f^	1.4	0.9-2.3	0.171	1.6	0.9-3.0	0.095
SHI^c,g^	1.2	0.8-1.9	0.337	1.3	0.8-2.0	0.345
SRC^c^	1.2	0.4-3.0	0.765	1.5	0.5-4.2	0.425
ACA+ only (n = 898)	OR	95%CI	p	OR	95%CI	p
Age older than cohort median at SSc onset (N = 46.8 years^b^)	1.2	0.9-1.7	0.208	0.9	0.6-1.3	0.557
Male sex	0.6	0.3-1.1	0.087	1.1	0.6-2.1	0.681
PAH^c^	0.8	0.5-1.3	0.406	0.5	0.3-0.9	0.020
ILD^c,d^	2.1	1.4-3.3	0.001	1.5	0.9-2.5	0.117
Myositis^c,e^	4.1	1.9-9.0	< 0.001	5.4	2.5-11.9	< 0.001
Synovitis^c^	2.4	1.8-3.3	< 0.001	5.4	3.8-7.6	< 0.001
Gastrointestinal involvement^c,f^	1.6	0.7-3.5	0.257	1.5	0.6-4.0	0.403
SHI^c,g^	1.6	1.0-2.8	0.061	1.3	0.7-2.3	0.471
SRC^c^	1.5	0.4-5.1	0.508	1.2	0.3-5.3	0.830
Scl70+ only (n = 290)	OR	95%CI	p	OR	95%CI	p
Age older than cohort median at SSc onset (N = 46.8 years^b^)	0.7	0.4-1.1	0.229	0.4	0.2-0.7	0.001
Male sex	0.8	0.4-1.4	0.374	1.1	0.6-2.0	0.843
PAH^c^	7.8	1.9-31.9	0.004	0.9	0.3-2.3	0.772
ILD^c,d^	2.0	1.2-3.5	0.010	1.9	1.1-3.4	0.030
Myositis^c,e^	2.1	0.7-6.2	0.194	11.0	1.3-90.5	0.026
Synovitis^c^	2.3	1.4-2.9	0.001	4.9	2.8-8.7	< 0.001
Gastrointestinal involvement^c,f^	9.8	1.8-53.9	0.009	1.6	0.5-5.4	0.412
SHI^c,g^	1.9	0.7-5.2	0.228	2.9	0.9-9.8	0.077
SRC^c^	4.2	0.4-42.8	0.230	3.0	0.2-37.9	0.399
RNAP3+ only (n = 195)	OR	95%CI	p	OR	95%CI	p
Age older than cohort median at SSc onset (N = 46.8 years^b^)	0.7	0.4-1.4	0.312	0.7	0.3-1.3	0.258
Male sex	0.9	0.4-1.8	0.683	0.9	0.4-2.0	0.738
Diffuse SSc	0.8	0.3-2.3	0.668	0.5	0.2-1.6	0.248
PAH^c^	3.2	1.6-6.4	0.001	4.4	2.0-9.7	< 0.001
ILD^c,d^	0.5	0.2-1.7	0.277	8.1	0.9-69.9	0.057
Myositis^c,e^	2.4	1.3-4.5	0.005	3.2	1.7-6.1	< 0.001
Synovitis^c^	0.3	0.1-1.9	0.220	0.5	0.1-3.2	0.494
Gastrointestinal involvement^c,f^	0.6	0.2-1.8	0.372	0.3	0.1-1.1	0.071
SHI^c,g^	2.3	0.9-6.0	0.097	1.4	0.5-3.8	0.515
SRC^c^	0.7	0.4-1.4	0.312	0.7	0.3-1.3	0.258

Abbreviations: dcSSc: diffuse cutaneous SSc; DMARD: disease-modifying anti-rheumatic drug; ILD: interstitial lung disease; lcSSc: limited cutaneous SSc; N: number; PAH: pulmonary arterial hypertension; SHI: SSc heart involvement; SSc: systemic sclerosis; SRC: SSc renal crisis.

a Denotes any use of either conventional synthetic or biologic DMARD ever.

b If age at SSc onset missing (N = 127), to avoid excluding participants grouped as older half of cohort if above median age at ASCS recruitment (57 years).

c Denotes ever from SSc onset.

d ILD diagnosed on HRCT.

e Myositis was identified by the treating clinician based on positive biopsy or elevated CK combined with abnormal MRI or EMG findings.

f Gastrointestinal symptoms defined as participants ever having upper GI involvement (ever reporting reflux symptoms, Barret’s oesophagus, oesophageal dysmotility, oesophageal stricture, dysphagia, vomiting or gastric antral vascular ectasia) or lower GI involvement (ever reporting bowel dysmotility, pseudo-obstruction, faecal incontinence, constipation, diarrhoea or bloating).

g SHI was determined by physician assessment based on the presence of systolic or diastolic dysfunction or rhythm disturbance attributable to SSc.

#### Subgroup analysis by disease subtype and autoantibody status

In both dcSSc and lcSSc, ILD and synovitis were associated with prednisolone and DMARD use (all p < 0.001; Table 3). Myositis was associated with use of both prednisolone and DMARDs in lcSSc (p < 0.001) and use of DMARDs (p = 0.012) but not prednisolone in dcSSc (p = 0.280). Age was inversely associated with DMARD use in both dcSSc and lcSSc (p ⩽ 0.039), while in lcSSc, only male sex was also associated with an increase in DMARD use, although not meeting statistical significance (p = 0.075). SHI was associated with only prednisolone use in dcSSc (p = 0.038). PAH was inversely associated with prednisolone use in lcSSc (p < 0.001), while it was associated with a small increase in prednisolone use in dcSSc, although not meeting statistical significance (p = 0.065). Gastrointestinal involvement was positively associated with prednisolone use in dcSSc (p = 0.089) and DMARD use in lcSSc (p = 0.095), although not meeting statistical significance.

In those with ACA positivity (Table 3), associations of prednisolone use were similar to those with lcSSc apart from SHI being associated with prednisolone use (p = 0.061). Myositis and synovitis were strongly associated with both prednisolone and DMARD use in those with ACAs (all p < 0.001), and PAH was inversely associated with DMARD use (p = 0.020). Among those with either Scl-70 or RNA polymerase-3 positivity, both prednisolone and DMARD use were associated with ILD and synovitis (p ⩽ 0.030). Myositis was also associated with DMARD but not prednisolone use in both groups (p ⩽ 0.057), as was SHI, although not meeting statistical significance (p ⩽ 0.077). Both PAH and gastrointestinal involvement were associated with prednisolone use in those with Scl-70 positivity only (p ⩽ 0.004), while age was inversely associated with DMARD use in those with Scl70 positivity only (p = 0.001).

### Changes in immunosuppressant prescribing over time

We compared patterns of early immunosuppressant use (prescription within 5 years of recruitment) in those with incident SSc recruited in two epochs (2007–2014 vs 2015–2024) (Table 4). Changes in medication availability for SSc in Australia during the study period are summarised in Supplementary Table S3. Immunosuppressants were more commonly prescribed in the later epoch (59% vs 42%, p < 0.001). This reflected an increase in DMARD use (56% vs 35%, p < 0.001), driven by an increase in csDMARDs (55% vs 34%, p < 0.001), although bDMARD use also increased (4% vs 1%, p = 0.020). There was a slight reduction in prednisolone use in the more recent epoch (17% vs 24%, p = 0.046), with those in this epoch receiving prednisolone therapy at fewer visits than those in the earlier epoch (median = 2 (IQR = 1–3) visits vs median = 3 (IQR = 1–4) visits, p < 0.01).

**Table 4.** Patterns of early immunosuppressant use (within 5 years of SSc onset) in those with incident SSc (n = 748) recruited in an earlier epoch (2007–2014, n = 507) or later epoch (2015–2024, n = 241).

Variable	Later epoch (2015–2024, n = 241)	Earlier epoch (2007–2014, n = 507)	p
Any immunosuppressant use^a,b^	141 (59%)	213 (42%)	< 0.001
Prednisolone^a^	42 (17%)	121 (24%)	0.046
Hydroxychloroquine^a^	37 (15%)	63 (12%)	0.272
Any DMARD^a,c^	136 (56%)	178 (35%)	< 0.001
Conventional synthetic DMARD^a,d^	132 (55%)	171 (34%)	< 0.001
Biologic agent^a,e^	9 (4%)	6 (1.2%)	0.020
IVIG^a^	8 (3%)	3 (0.6%)	0.004
Cyclophosphamide^a^	2 (0.8%)	8 (1.6%)	0.405

csDMARD: conventional synthetic disease-modifying anti-rheumatic drug; DMARD: disease-modifying anti-rheumatic drug; dcSSc: diffuse SSc; IVIG: intravenous immune globulin; lcSSc: limited SSc; n: number; TNF: tumour necrosis factor.

Complete data available for all variables (n = 748) unless otherwise specified.

a Denotes ever from SSc onset.

b Denotes use of any glucocorticoid or non-glucocorticoid immunosuppressant, including biologics, IVIG and cyclophosphamide.

c Denotes any use of either conventional synthetic or biologic DMARD ever.

d Conventional synthetic DMARDs include azathioprine, methotrexate, mycophenolate, calcineurin inhibition and leflunomide.

e Biologic agents include abatacept, TNF-alpha inhibitors, tocilizumab or B-cell depletion.

The time from SSc onset to immunosuppressant exposure was compared in those with early SSc (<5 years SSc onset to recruitment) where the first ASCS visit occurred between 2007 and2014 versus 2015 and2024. DMARDs were commenced earlier in those recruited between 2015 and 2024 (1.8 (IQR = 1.0–3.2) years versus 2.4 (IQR = 1.2–4.0) years, p = 0.011) despite no difference in SSc duration at recruitment (1.6 (0.9–2.7) years versus 1.7 (0.9–2.9) years, p = 0.376).

## Discussion

In this large, longitudinal SSc cohort study, we have reported the frequency and clinical associations of use of immunosuppressants in over 2000 individuals. Sixty percent of this cohort received immunosuppressants, including over 80% of those with dcSSc compared to 50% of those with lcSSc. This reflects an increase from previous studies and other large cohorts, which have demonstrated that around 40% of SSc patients may receive immunosuppressants, with around 60% of dcSSc and 30% of lcSSc receiving immunosuppressants.^1,2^ Among our cohort overall, almost half received prednisolone, and 40% other DMARDs. We found that DMARD prescription appears to be increasing in SSc, while glucocorticoid use is reduced. Unsurprisingly, severe or inflammatory features of SSc, dcSSc and internal organ involvement were associated with immunosuppressant use. Overall, these data provide insights into the determinants of immunosuppressants including in clinical and serological subgroups, and the changing landscape of SSc treatment with the introduction and increased accessibility of newer treatments.

We found that in incident SSc, immunosuppressants are being started earlier in the disease course. This finding reflects changing treatment recommendations in SSc, for example, the increased and earlier use of mycophenolate after its recommendation for SSc-ILD and equivalent efficacy to cyclophosphamide, as well as the recommendation for skin fibrosis.^2,7,12^ This trend also in part reflects increased access to certain agents in the Australian context, with mycophenolate available on the Australian Pharmaceutical Benefits Scheme for only limited indications until 2015. This is particularly true for biologic agents which were generally infrequently prescribed in our cohort. In Australia, access to biologic agents has relied on hospital-funding or an additional funded indication (e.g. concomitant rheumatoid arthritis) or compassionate/hospital-funded supply until 2022, when access to rituximab became unrestricted. Furthermore, several agents commonly prescribed in our cohort, particularly hydroxychloroquine, are not represented in recent SSc treatment guidelines due to a lack of a robust evidence base, although their use is supported by expert opinion.^7,12^ Hydroxychloroquine in particular may be prescribed for arthralgia/arthritis in SSc with a low risk of toxicity and favourable data in a small retrospective series.^13^ Use of hydroxychloroquine is thought to be widespread, although the precise frequency is not well-quantified in other cohorts. The use of non-evidence-based agents reflects a historic lack of SSc-specific treatment options, with treatment for several manifestations, particularly arthritis, extrapolated from other rheumatological conditions. However, overall, these data demonstrate both an increased and earlier use of DMARDs in SSc, in line with changing treatment recommendations.^7,14^

However, while glucocorticoid use appears to have reduced overtime in SSc, these data highlight the fact that over 40% of this real-world SSc cohort received glucocorticoids, despite potential toxicities including the association with SRC.^5^ There is a paucity of evidence supporting the use of glucocorticoids in SSc, which may be prescribed for symptom control in inflammatory musculoskeletal manifestations and also historically for SSc-ILD.^15,16^ Data in other cohorts suggest a high frequency of glucocorticoid prescribing in other real-world cohorts, particularly in early dcSSc with rates of over 40%–50%.^17,18^ While expert opinion supports a role for symptom control in inflammatory musculoskeletal SSc features,^12,15,16^ evidence in fact suggests no additional role for glucocorticoids in SSc-ILD,^19^ leading to recent recommendations against the use of glucocorticoids in SSc-ILD.^20^ Treatment guidelines suggest close monitoring of renal function and blood pressure with glucocorticoid treatment due to the risk of SRC, particularly in high-risk groups, for example, those with dcSSc or RNA polymerase 3 positivity.^7,14^ Reassuringly, we did not find a specific association of prednisolone use with SRC in those with dcSSc or RNA polymerase-3 positivity, albeit with small numbers in some subgroups. We were not able to quantify glucocorticoid doses, as the ASCS does not collect the dose or precise duration of treatment with individual agents, so we were not able to assess any change in glucocorticoid dosing over time. However, we identified that those in the more recent epoch were exposed to prednisolone at fewer visits than those recruited in the earlier epoch. This supports findings from the EUSTAR cohort suggesting a reduction in glucocorticoid prescribing over time.^17^ It is hoped that easier and earlier access to non-glucocorticoid immunosuppressants, particularly bDMARDs, will continue to reduce the need for glucocorticoids in SSc, as has been the case with SSc-ILD, where the emergence of effective non-glucocorticoid treatment options has led to a recommendation against glucocorticoid use.^20^

It has been previously recognised that those with dcSSc are more likely to require immunosuppressants than lcSSc.^1,2^ In our cohort, ILD, myositis and synovitis were also generally consistent associations of immunosuppressant exposure. Myositis was not identified as an association of glucocorticoid use in dcSSc; this may reflect a lack of statistical power rather than a true lack of association, as myositis was uncommon in our cohort. Limited data describe the associations of immunosuppressant use in different SSc subgroups, particularly outside of early dcSSc. Broadly, the associations of immunosuppressant use were similar across subgroups and autoantibody profile, particularly in the case of lcSSc and ACA positivity. However, PAH was associated with prednisolone exposure in dcSSc and Scl-70 positivity, likely reflecting the fact that most of these individuals had concomitant PAH and ILD rather than a direct association with PAH itself. Overall, these data suggest that while immunosuppressant use is more common in dcSSc, the determinants of use are broadly similar between both disease subtypes and autoantibody profiles.

This study has limitations. The ASCS does not collect detailed data regarding the dose and duration of use of immunosuppressive agents, with information recorded as current/never/previous use at each annual review. Furthermore, information regarding indications for treatment is not collected, which means we cannot examine the direct impact of changing treatment recommendations for certain SSc features over time, for example, ILD or worsening skin involvement. Treatment data in this prospective observational study are subject to confounding by indication. Use of certain agents which are given episodically as infusions (e.g. cyclophosphamide) may be underestimated if the patient was not seen at the time of their treatment course and therefore not recorded in a participant’s record. As study visits are conducted annually, it is possible that short courses of treatment prescribed between visits were missed or listed only as ‘previous’ treatment exposure rather than ‘current’. This may have limited the accuracy of our analysis comparing treatment epochs, as only current use was captured. Finally, the small number of patients with dcSSc and certain complications such as myositis limit the power of some analyses, underestimating the associations among these variables.

## Conclusion

Immunosuppressants are commonly prescribed for individuals with SSc, with 60% of participants receiving immunosuppressants including over 80% of those with dcSSc and 50% of those with lcSSc. Those requiring immunosuppressants had severe or inflammatory SSc features, irrespective of the extent of skin involvement or serological profile. These real-world data demonstrate that the frequency of non-glucocorticoid immunosuppressant use in SSc is increasing over time, and it is being commenced earlier in the disease course. Reassuringly, exposure to prednisolone also seems to be reducing over time.

## Supplemental Material

sj-pdf-1-jso-10.1177_23971983251342690 – Supplemental material for Frequency and determinants of use of immunosuppressants in the Australian Scleroderma Cohort Study Supplemental material, sj-pdf-1-jso-10.1177_23971983251342690 for Frequency and determinants of use of immunosuppressants in the Australian Scleroderma Cohort Study by Jessica L Fairley, Dylan Hansen, Susanna Proudman, Joanne Sahhar, Gene-Siew Ngian, Diane Apostolopoulos, Jennifer Walker, Lauren V Host, Wendy Stevens, Laura Ross and Mandana Nikpour in Journal of Scleroderma and Related Disorders
