# Supplementary material for: Frequency and determinants of use of immunosuppressants in the Australian Scleroderma Cohort Study
Source: J Scleroderma Relat Disord. 2025 May 22;10(3):311–21. doi: 10.1177/23971983251342690 (PMC12102085; doi:10.1177/23971983251342690)
Supplement: sj-pdf-1-jso-10.1177_23971983251342690 – Supplemental material for Frequency and determinants of use of immunosuppressants in the Australian Scleroderma Cohort Study [file sj-pdf-1-jso-10.1177_23971983251342690.pdf]

Supplementary Table S1: Clinical features of those receiving immunosuppression by disease duration at recruitment (n=1149).

Variable[&]	Longer SSc duration ≥7 years at recruitment (n=540, 47.0%)	Shorter SSc duration<7 years at recruitment (n=609, 53.0%)	p-value
**Age at SSc onset (years)**	41.4 (31.8-50.6)	52.2 (42.4-61.3)	<0.001
**Male sex**	56 (10.4%)	135 (22.2%)	<0.001
**Diffuse cutaneous SSc**	146 (27.0%)	276 (45.3%)	<0.001
**Non-Caucasian Ethnicity (n=1812)**	41 (7.8%)	73 (12.4%)	0.010
**Disease duration at recruitment (years)**	15.5 (10.6-22.8)	2.2 (1.1-4.2)	<0.001
**Follow-up (years)**	4.9 (1.8-9.1)	5.2 (2.0-92)	0.419
**Overlap features (n=765)**	76 (22.5%)	66 (15.5%)	0.013
Inflammatory arthritis (n=765)	33 (9.8%)	34 (8.0%)	0.382
SLE (n=765)	14 (4.1%)	11 (2.6%)	0.226
Polymyositis/Dermatomyositis (n=765)	13 (3.9%)	16 (3.8%)	0.943
Sjogren Syndrome (n=765)	24 (7.1%)	10 (2.3%)	0.002
**ANA Centromere* (n=1114)**	223 (42.1%)	146 (25.0%)	<0.001
**ENA***			
Scl70 (n=1105)	79 (15.1%)	141 (24.2%)	<0.001
Ro (n=1107)	52 (9.9%)	72 (12.4%)	0.194
La (n=1104)	8 (1.5%)	17 (2.9%)	0.117
U1RNP (n=1104)	47 (9.0%)	51 (8.8%)	0.903
Scl/PM (n=1102)	9 (1.7%)	23 (4.0%)	0.027
**RNA polymerase-3* (n=846)**	52 (13.5%)	86 (18.7%)	0.044
**Ischaemic heart disease[1]* (n=1140)**	100 (18.7%)	77 (12.7%)	0.005
**SHI*[2]**	59 (10.9%)	72 (11.8%)	0.633
**LVEF<50%* (n=1020)**	32 (6.8%)	38 (7.0%)	0.895
**PAH***	76 (14.1%)	54 (8.9%)	0.005
**ILD (on HRCT)*[3]**	194 (35.9%)	232 (38.1%)	0.447
**Digital ulcers***	317 (58.7%)	334 (54.8%)	0.188

**Raynaud's Phenomenon***	536 (99.3%)	602 (98.9%)	0.478
**Tendon Friction Rubs* (n=1132)**	50 (9.4%)	81 (13.5%)	0.030
**Synovitis* (n=1142)**	280 (52.2%)	334 (55.1%)	0.331
**SRC***	21 (3.9%)	29 (4.8%)	0.469
**Gastrointestinal involvement*[4]**	517 (95.7%)	582 (95.6%)	0.885
**Myositis*[5]**	54 (10.0%)	75 (12.3%)	0.215
**CRP>5mg/L* (n=1113)**	324 (62.0%)	347 (58.8%)	0.286

Data presented for whole cohort (n=1149) unless otherwise specified. **Abbreviations:** ANA (antinuclear antibody), CK (creatine kinase), CRP (C-reactive protein) dcSSc (diffuse cutaneous systemic sclerosis), DLCO (diffusing capacity for carbon monoxide), DMARD (disease modifying antirheumatic drug), ENA (extractable nuclear antigen), FVC (forced vital capacity), ILD (interstitial lung disease), IVIG (intravenous immune globulin), LVEF (left ventricular ejection fraction), mm/hr (millimetres per hour), mmHg (millimetres of mercury), mg/L (milligram per litre), MRSS (Modified Rodnan Skin Score), PAH (pulmonary arterial hypertension), RVSP (right ventricular systolic pressure), SHI (SSc Heart Involvement), SSc (systemic sclerosis), TNF (tumour necrosis factor).

[&]Number and percentage or median/interquartile range presented as appropriate. *Ever from SSc onset. ^If value at first visit not available, first recorded value within 5 years of recruitment presented. [#]Immunosuppressant exposure defined as any immunosuppressive or immunomodulatory therapy including corticosteroids, IVIg, synthetic or biologic disease-modifying therapy. [1]Ischaemic heart disease defined as patient-reported angina/myocardial infarction, or abnormal coronary angiography. [2]SHI was determined by physician assessment based on the presence of systolic or diastolic dysfunction or rhythm disturbance attributable to SSc. [3]ILD diagnosed on high-resolution CT (HRCT) chest. [4]Gastroinestinal involvement defined as ever experiencing upper (reflux symptoms, Barret's oesophagus, oesophageal dysmotility, oesophageal stricture, dysphagia, vomiting or gastric antral vascular ectasia) or lower gastrointestinal involvement (bowel dysmotility, pseudo-obstruction, faecal incontinence, constipation, diarrhoea, or bloating). [5]Myositis was identified by the treating clinician based on positive biopsy or elevated CK combined with abnormal MRI or EMG findings.

Supplementary Table S2: Univariable logistic regression analysis for the associations of exposure to immunosuppressants.

Variable	Odds Ratio	95% Confidence Interval	p-value
**Cohort overall**			
**Associations of prednisolone exposure**			
Age older than cohort median at SSc onset (N=46.8 years[1])	0.9	0.7-1.0	0.147
Male sex	1.3	1.0-1.6	0.054
dcSSc	2.2	1.8-2.7	<0.001
PAH*	1.1	0.9-1.5	0.396
ILD*[2]	2.4	2.0-3.0	<0.001
Myositis*[3]	3.6	2.5-5.3	<0.001
Synovitis*	2.3	1.9-2.8	<0.001
Gastrointestinal involvement*[4]	1.8	1.2-2.7	0.004
SHI*[5]	1.8	1.3-2.4	<0.001
SRC*	1.9	1.2-3.1	0.009
**Associations of DMARD exposure[6]**			
Age older than cohort median at SSc onset (N=46.8 years[1])	0.6	0.5-0.7	<0.01
Male sex	1.4	1.1-1.8	0.004
dcSSc	3.3	2.7-4.0	<0.001
PAH*	0.6	0.5-0.8	0.002
ILD*[2]	2.4	2.0-2.9	<0.001
Myositis*[3]	5.0	3.4-7.3	<0.001
Synovitis*	4.0	3.3-4.9	<0.001
Gastrointestinal involvement*[4]	2.0	1.3-3.1	0.001
SHI*[5]	1.5	1.1-2.1	0.006
SRC*	1.7	1.1-2.8	0.023

*Denotes ever from SSc onset. [1]If age at SSc onset missing (N=127), to avoid excluding participants grouped as older half of cohort if above median age at ASCS recruitment (57 years). [2]ILD diagnosed on HRCT. [3]Myositis was identified by the treating clinician based on positive biopsy or elevated CK combined with abnormal MRI or EMG findings. [4]Gastrointestinal symptoms defined as participants ever having upper GI involvement (ever reporting reflux symptoms, Barret's oesophagus, oesophageal dysmotility, oesophageal stricture, dysphagia, vomiting or gastric antral vascular ectasia) or lower GI involvement (ever reporting bowel dysmotility, pseudo-obstruction, faecal incontinence, constipation, diarrhoea, or bloating). [5]SHIwas determined by physician assessment based on the presence of systolic or diastolic dysfunction or rhythm disturbance attributable to SSc. [6]DMARD exposure includes any conventional synthetic or biologic DMARD. **Abbreviations**: ACA (anticentromere antibody), dcSSc (diffuse cutaneous SSc), DMARD (disease-modifying anti-rheumatic drug), ILD (interstitial lung disease), lcSSc (limited cutaneous SSc), N (number), PAH (Pulmonary arterial hypertension), SHI (SSc Heart Involvement), SSc (systemic sclerosis), SRC (SSc Renal Crisis).

Supplementary Table S3: Medication Availability in Australia over time during the study period (2007 to December 2024).

Agent	Availability
**Prednisolone**	Pre-2007
**Hydroxychloroquine**	Pre-2007
**Azathiorpine**	Pre-2007
**Calcineurin inhibitors (cyclosporine, tacrolimus)**	Pre-2007
**Leflunomide**	Pre-2007
**Methotrexate**	Pre-2007
**Cyclophosphamide**	Pre-2007
**Mycophenolate**	Pre 2015: First available for use in transplant patients from around 1997, subsequently listed for treatment of lupus nephritis only from around 2010. Compassionate or hospital-funded supply potentially available depending for individuals with SSc on a case-by-case basis.<br>2015 onwards: available on the PBS for unrestricted indications, meaning widely available for use in SSc.
**Abatacept**	Not specifically available on the PBS for SSc; available for the treatment of rheumatoid arthritis only. Compassionate or hospital-funded supply potentially available depending for individuals with SSc on a case-by-case basis.
**TNF-alpha inhibitors**	Not specifically available on the PBS for SSc; available for other indications including the treatment of inflammatory arthritis (rheumatoid or psoriatic), inflammatory bowel disease or skin psoriasis. Compassionate or hospital-funded supply potentially available depending for individuals with SSc on a case-by-case basis.
**Tocilizumab**	Not specifically available on the PBS for SSc; available for the treatment of rheumatoid arthritis only. Compassionate or hospital-funded supply potentially available depending for individuals with SSc on a case-by-case basis.
**Rituximab**	Pre 2021: available only for use in pre-specified indications e.g., rheumatoid arthritis. Compassionate or hospital-funded supply potentially available depending for individuals with SSc on a case-by-case basis.<br>2021 onwards: unrestricted prescription of rituximab on the PBS facilitates access to treatment for SSc.

Abbreviations: TNF (tumour necrosis factor).
